# Metabolic clearance rate of insulin across the glucose tolerance spectrum by race and ethnicity in youth with obesity

**DOI:** 10.1002/oby.24317

**Published:** 2025-06-05

**Authors:** Wonhee Cho, Fida Bacha, Hala Tfayli, SoJung Lee, Sara F. Michaliszyn, Joon Young Kim, Silva Arslanian

**Affiliations:** ^1^ Department of Exercise Science, David B. Falk College of Sport and Human Dynamics Syracuse University Syrascuse New York USA; ^2^ Children's Nutrition Research Center Baylor College of Medicine Houston Texas USA; ^3^ Department of Pediatrics and Adolescent Medicine American University of Beirut Medical Center Beirut Lebanon; ^4^ Division of Sports Medicine and Science, Graduate School of Physical Education Kyung Hee University Yongin Republic of Korea; ^5^ Department of Kinesiology and Sport Science Youngstown State University Youngstown Ohio USA; ^6^ Center for Pediatric Research in Obesity and Metabolism and Division of Pediatric Endocrinology University of Pittsburgh, School of Medicine, UPMC Children's Hospital of Pittsburgh Pittsburgh Pennsylvania USA

## Abstract

**Objective:**

Despite β‐cell failure in youth with dysglycemia (i.e., impaired glucose tolerance [IGT] and type 2 diabetes), fasting insulin (FI) concentrations are elevated. Herein, we examined the following: 1) metabolic clearance rate of insulin (MCRI) in youth with obesity and normal glucose tolerance (NGT) versus those with IGT versus those with type 2 diabetes; 2) racial and ethnic differences in insulin dynamics; and 3) metabolic/adiposity correlates of MCRI.

**Methods:**

A total of 206 youth underwent assessment of fasting glucose, FI, MCRI and peripheral insulin sensitivity (PIS), first‐phase insulin secretion, disposition index, body composition, and abdominal adiposity.

**Results:**

In type 2 diabetes versus NGT, MCRI was lower (*p* < 0.001), and FI was higher (*p* < 0.001). In Black versus White youth, MCRI was lower (*p* < 0.001), driven by lower MCRI in youth with dysglycemia (*p* < 0.001) and not with NGT. MCRI correlated inversely with FI, as well as adiposity measures, and correlated directly with PIS and disposition index. Lower PIS, lower MCRI, and higher first‐phase insulin secretion were characteristics of Black versus White youth with dysglycemia.

**Conclusions:**

Higher FI concentrations in the presence of dysglycemia despite β‐cell failure could be explained by decreased MCRI. Racial and ethnic contrast in insulin dynamics differs by glycemic status and is more pronounced in dysglycemia manifested by lower MCRI and heightened first‐phase insulin secretion in Black versus White youth.

**Study Importance**

**What is already known?**

- Fasting insulin concentrations are elevated despite β‐cell failure in youth with obesity and dysglycemia (impaired glucose tolerance [IGT] and type 2 diabetes).

**What does this study add?**

- There is a significant reduction in metabolic clearance rate of insulin (MCRI) in youth with obesity and type 2 diabetes compared with their peers with normal glucose tolerance, correlating inversely with fasting insulin concentrations and adiposity and correlating directly with peripheral insulin sensitivity and β‐cell function.
- Black youth with IGT and type 2 diabetes, compared with their White peers, have lower MCRI, with a metabolic profile characterized by upregulated first‐phase insulin.

**How might these results change the direction of research or the focus of clinical practice?**

- Race‐ and ethnicity‐specific differences in MCRI may play an important role in the regulation of insulin dynamics and glucose homeostasis in youth with obesity.

## INTRODUCTION

Youth‐onset type 2 diabetes has increased dramatically in the United States, with a 97.1% increase in prevalence between 2001 and 2017 [1] and an annual increase in incidence by 5.3% between 2002 and 2018 [2]. In line with type 2 diabetes in adults, impaired β‐cell function in the presence of insulin resistance represents two major pathophysiological components of impaired glucose tolerance (IGT) and type 2 diabetes in youth [3, 4], although the severity of insulin resistance in youth is worse than that in adults [5, 6, 7]. We previously demonstrated that youth with obesity and type 2 diabetes compared with their normoglycemic peers have higher fasting insulin concentrations, despite significantly impaired β‐cell function [8]. The Restoring Insulin Secretion (RISE) study found that youth with recently diagnosed diabetes and IGT compared with their adult counterparts have lower insulin clearance, assessed by the ratio of fasting C‐peptide to fasting insulin, in the presence of greater β‐cell function [6]. Moreover, in an earlier study of ours, we found that normal‐weight, normoglycemic Black youth have lower insulin clearance in the presence of not only higher fasting but also higher stimulated first‐phase insulin concentrations compared with their White peers [9]. Against this backdrop, we hypothesized that a potential explanation for elevated fasting insulin concentrations with impaired β‐cell function in youth with type 2 diabetes [8] could be decreased metabolic clearance rate of insulin (MCRI). On the other hand, race‐ and ethnicity‐related differences in insulin dynamics and upregulated β‐cell function in Black versus White youth [9, 10] could underlie the observed finding of heightened β‐cell function and lower MCRI in Black versus White youth [9]. Therefore, in trying to tease out the relationships among MCRI (using the hyperinsulinemic‐euglycemic clamp), fasting insulin concentrations, and β‐cell function (using the hyperglycemic clamp), we examined the following: 1) MCRI in youth with obesity across the glucose tolerance spectrum from normal glucose tolerance (NGT) to IGT to type 2 diabetes; 2) racial and ethnic contrast in MCRI; and 3) the metabolic and adiposity correlates of MCRI by race and ethnicity in youth with obesity.

## METHODS

### Participants

A total of 206 participants (aged 10–20 years; Tanner stages II–V; 95 Black and 111 White; 102 male and 104 female individuals) from our National Institutes of Health (NIH)‐funded K24 grant investigating childhood insulin resistance are included, some of whom were included in previous publications unrelated to the current investigation [3, 11, 12, 13, 14]. There were 19 youth with overweight (body mass index [BMI] ≥ 85th but <95th percentile for age and sex) and 187 youth with obesity (BMI ≥ 95th percentile). For simplicity purposes, we will refer to all participants in the present study as having “obesity.” Among them, there were 138 participants with NGT, 36 with IGT, and 32 with type 2 diabetes. Glucose tolerance was determined with a 2‐h oral glucose tolerance test (OGTT; 1.75 g glucose/kg [maximum 75 g]) [15]. All youth with type 2 diabetes were negative for glutamic acid decarboxylase and insulinoma‐associated protein‐2 autoantibodies, and all had adequate metabolic control, with a mean (SE) hemoglobin A1c (HbA1c) of 5.5% (0.1%) (range 4.3%–8.3%) and mean (SE) duration of diabetes of 7.5 (1.7) months (range 0–39 months). They were on treatment consisting of lifestyle modification (*n* = 7), metformin alone (*n* = 15), metformin together with insulin (*n* = 7), and insulin alone (*n* = 3). Participants categorized as having NGT or IGT were not on any medications that could influence glucose metabolism.

### Study procedures

Participants were recruited via newspaper advertisements and fliers posted on the medical campus, buses, and the outpatient clinics in the Weight Management and Wellness Center and the Division of Pediatric Endocrinology at UPMC Children's Hospital of Pittsburgh in Pennsylvania. This study was approved by the institutional review board of the University of Pittsburgh, and written informed parental consent and child assent were obtained before any research procedures were performed in accordance with the ethics guidelines of Children's Hospital of Pittsburgh. All procedures were performed at the Pediatric Clinical and Translational Research Center of Children's Hospital of Pittsburgh. All participants were screened for medical history and underwent a physical examination with assessment of pubertal Tanner staging [16], as well as hematological and biochemical tests. Height and weight were assessed to the nearest 0.1 cm and 0.1 kg, respectively, and were used to compute BMI. Dual‐energy x‐ray absorptiometry was used to evaluate body composition, total body fat mass, and percentage of body fat. Visceral adipose tissue (VAT) was assessed via either computed tomography or magnetic resonance imaging (MRI) at the L4‐L5 intervertebral space [17, 18]. Note that Klopfenstein et al. [19] reported that there is strong correlation (*r* = 0.89–0.95) and good agreement between computed tomography and MRI in quantitating the abdominal adipose tissue.

### Metabolic studies

All participants were admitted the day prior to the clamp and twice randomly within a 1‐ to 4‐week period to the Pediatric Clinical and Translational Research Center for a hyperinsulinemic‐euglycemic clamp to assess in vivo peripheral insulin sensitivity (PIS) and MCRI and a hyperglycemic clamp to assess insulin secretion. Each clamp was conducted in the morning after a 10‐ to 12‐h overnight fast. PIS was evaluated during a 3‐h hyperinsulinemic (80 mU/m^2^/min)‐euglycemic (100 mg/dL) clamp. Plasma glucose was clamped at ~100 mg/dL with a variable rate infusion of 20% dextrose in water. The glucose infusion was adjusted based on arterialized plasma glucose measurements every 5 min, and blood was sampled every 10 to 15 min for determination of insulin. First‐phase insulin secretion was assessed during a 2‐h hyperglycemic (225 mg/dL) clamp. Plasma glucose was increased rapidly to 225 mg/dL by a bolus infusion of dextrose and maintained at that level by a variable rate infusion of 20% dextrose for 2 h, with frequent measurement of glucose concentrations (every 2.5–5 min). Insulin concentrations were measured at 2.5‐min intervals during the initial 15 min, followed by 15‐min intervals until the conclusion of the 120‐min clamp testing. Before the start of the clamp, fasting blood samples were obtained for total cholesterol, triglycerides, high‐density lipoprotein (HDL) cholesterol, low‐density lipoprotein (LDL) cholesterol, glucose, insulin, and HbA1c. In participants with type 2 diabetes, treatment (metformin and long‐acting insulin) was discontinued 48 h before study procedures.

### Biochemical measurements

Plasma glucose and insulin concentrations were determined using the glucose‐oxidase method in a glucose analyzer (Yellow Springs Instrument Co.) and using a commercially available radioimmunoassay (RIA) kit specific to human insulin (catalog no. HI‐14 K; LINCO Research, Inc. and MilliporeSigma), respectively. Leptin and adiponectin were assessed by a commercially available RIA kit (LINCO Research). Plasma lipid concentrations were determined using the standards of the Centers for Disease Control and Prevention [3].

### Calculations

MCRI, expressed in milliliters per meters squared per minute, was calculated by dividing insulin infusion rate (80 mU/m^2^/min) by the delta increase in circulating insulin concentrations from baseline to the clamp steady state (the last 30 min of the clamp) [20]. Insulin‐stimulated glucose disposal rate during the clamp was calculated as the mean exogeneous glucose infusion rate during the last 30 min of the clamp. Peripheral insulin sensitivity was calculated by dividing glucose disposal rate by the steady‐state clamp insulin concentration multiplied by 100 (expressed in milligrams per meters squared per minute per microunits per milliliter) [21]. First‐phase insulin was calculated during the 2‐h hyperglycemic clamp for the times at 2.5, 5, 7.5, 10, and 12.5 min, as has been described earlier [9]. Disposition index (DI), which represents insulin secretion relative to insulin sensitivity, was calculated as the product of PIS and first‐phase insulin secretion [3, 11, 12]. Fasting glucose and insulin concentrations were calculated as the average of two measurements taken from the hyperinsulinemic‐euglycemic and hyperglycemic clamps, with a range of 0.02% difference in glucose and 6.8% difference in insulin concentrations between the two clamps.

### Statistical analysis

Independent‐samples *t* tests, χ^2^ tests, ANCOVA, all adjusting for age, sex, race, Tanner stage, and BMI, analyses were conducted to compare MCRI and physical and metabolic characteristics across glycemic status (NGT vs. IGT vs. type 2 diabetes) and race (Black vs. White). Pearson correlation analysis was performed to examine the relationships between MCRI and metabolic and adiposity variables. Variables that did not meet normality assumptions were log_10_‐transformed, with untransformed data being presented for ease of interpretation. Data are presented as mean (SE) with *p* ≤ 0.05.

## RESULTS

### Physical and metabolic characteristics in youth with obesity across the glucose tolerance spectrum

There were no differences in race, sex, percent body fat, total cholesterol, LDL cholesterol, and leptin among the three groups (Table 1). Youth with IGT and type 2 diabetes versus those with NGT were slightly older and had higher weight and VAT (Table 1). Youth with type 2 diabetes versus those with NGT had advanced Tanner stage, lower HDL cholesterol, lower adiponectin, and higher fasting insulin (Table 1). Youth with type 2 diabetes versus those with NGT and IGT had higher HbA1c and fasting glucose and lower first‐phase insulin, with a progressive decrease in PIS and DI moving across groups of glucose tolerance from NGT to IGT to type 2 diabetes (Table 1). All statistical differences in lipid and metabolic parameters, except for HDL cholesterol, remained significant after adjustment for age, sex, race, Tanner stage, and BMI (Table 1).

**TABLE 1:** Physical and metabolic characteristics of the three groups of youth with obesity, i.e., those with Ob‐NGT, Ob‐IGT, and Ob‐T2D.

	Ob‐NGT (1), *n* = 138	Ob‐IGT (2), *n* = 36	Ob‐T2D (3), *n* = 32	*p*_ANOVA_	*p* value, post hoc		
					1 vs. 2	1 vs. 3	2 vs. 3
Physical characteristics							
Age, y	14.0 ± 0.2	15.0 ± 0.3	15.0 ± 0.3	0.002	0.016	0.023	NS
Race, % Black/White	48/52	36/64	50/50	NS			
Sex, % M/F	51/49	47/53	44/56	NS			
Tanner stage, % II–III/IV–V	35/65	17/83	9/91	0.004	NS	<0.001	NS
Weight, kg	91.1 ± 2.0	102.1 ± 3.5	102.4 ± 3.6	0.004	0.03	0.03	NS
BMI, kg/m^2^	33.8 ± 0.6	36.5 ± 1.1	36.4 ± 0.9	0.02	NS	NS	NS
BMI *z* score	2.2 ± 0.03	2.3 ± 0.1	2.4 ± 0.1	0.048	NS	NS	NS
Fat mass, kg	37.3 ± 1.1	44.9 ± 2.1	40.9 ± 1.8	0.004	0.004	NS	NS
Percent body fat, %	41.7 ± 0.7	44.4 ± 0.9	42.5 ± 1.2	NS			
VAT, cm^2^	66.7 ± 3.0	77.5 ± 4.6	86.8 ± 7.3	0.001*	0.048	0.004*	NS
Fasting‐based metabolic parameters							
Total cholesterol, mg/dL	157.8 ± 2.8	165.5 ± 6.2	157.4 ± 5.6	NS			
Triglycerides, mg/dL	108.8 ± 4.5	137.3 ± 14.6	135.9 ± 12.3	0.019*	NS	NS	NS
HDL cholesterol, mg/dL	41.5 ± 0.7	38.4 ± 1.3	37.7 ± 1.4	0.012	NS	0.04	NS
LDL cholesterol, mg/dL	94.7 ± 2.4	100.3 ± 5.4	92.7 ± 5.1	NS			
HbA1c, %	5.3 ± 0.1	5.3 ± 0.1	6.6 ± 0.1	<0.001*	NS	<0.001*	<0.001*
Fasting glucose, mg/dL	95.7 ± 0.5	96.4 ± 1.3	121.0 ± 4.0	<0.001*	NS	<0.001*	<0.001*
Fasting insulin, μU/mL	32.9 ± 1.2	40.0 ± 3.2	54.8 ± 8.5	<0.001*	NS	<0.001*	NS
Leptin, ng/mL	33.9 ± 1.2	38.9 ± 3.8	31.5 ± 2.5	NS			
Adiponectin, μg/mL	7.6 ± 0.3	6.8 ± 0.5	5.2 ± 0.5	0.005*	NS	0.004*	NS
Clamp‐derived parameters							
PIS, mg/m^2^/min per μU/mL	124.2 ± 7.6	89.4 ± 9.1	67.1 ± 8.2	<0.001*	0.007*	<0.001*	0.05*
First‐phase insulin, μU/mL	229.9 ± 14.7	217.7 ± 20.7	123.1 ± 26.6	<0.001*	NS	<0.001*	<0.001*
Clamp DI, mg/m^2^/min	24,283.9 ± 1466.9	15,120.7 ± 1391.6	5308.7 ± 552.3	<0.001*	0.017	<0.001*	<0.001*

*Note*: Data are presented as mean ± SE.

Abbreviations: DI, disposition index; F, female; HbA1c, hemoglobin A1c; HDL, high‐density lipoprotein; IGT, impaired glucose tolerance; LDL, low‐density lipoprotein; M, male; NGT, normal glucose tolerance; Ob, obesity; NS, not significant; PIS, peripheral insulin sensitivity; T2D, type 2 diabetes; VAT, visceral adipose tissue.

* *p* < 0.05 after adjustment for age, sex, race, Tanner stage, and BMI.

### MCRI in youth with obesity across the glucose tolerance spectrum and by race

**FIGURE 1:**
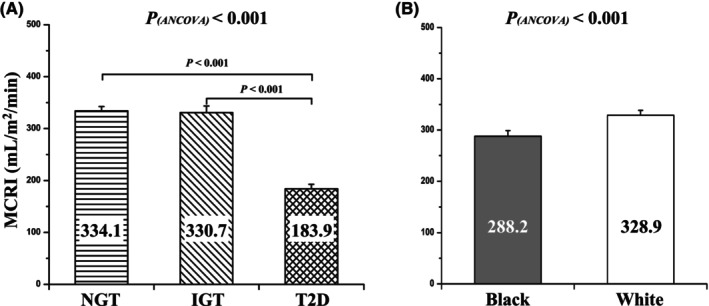
Differences in MCRI in youth with obesity. (A) NGT versus IGT versus T2D and (B) Black versus White. IGT, impaired glucose tolerance; MCRI, metabolic clearance rate of insulin; NGT, normal glucose tolerance; T2D, type 2 diabetes.

**FIGURE 2:**
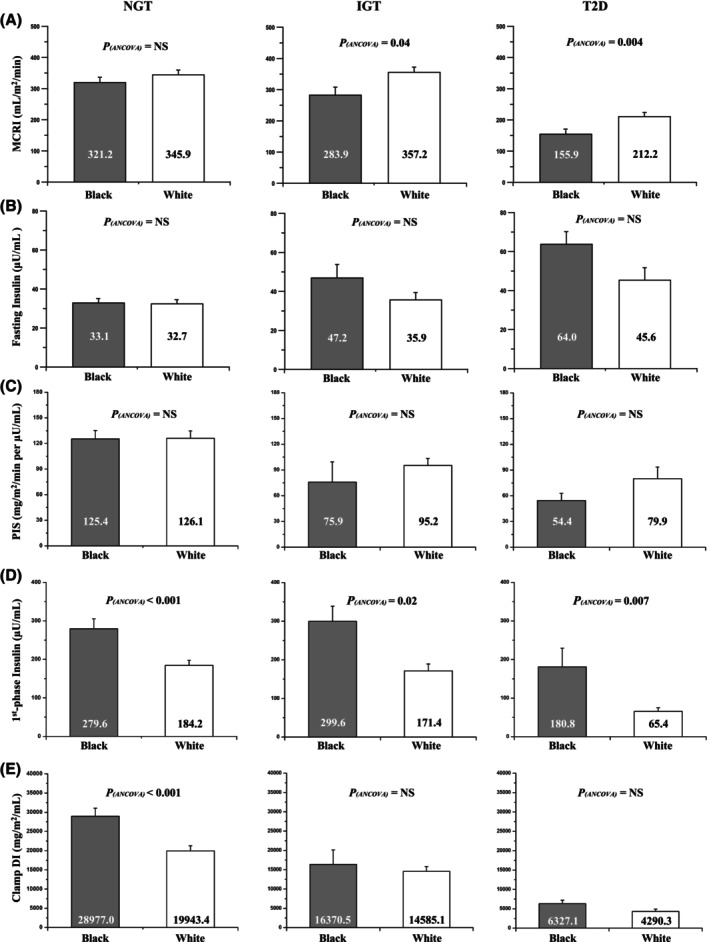
Differences in clamp‐derived metabolic characteristics between Black versus White youth with obesity in each glucose tolerance category (i.e., NGT, IGT, and T2D): (A) MCRI, (B) fasting insulin, (C) PIS, (D) first‐phase insulin, and (E) clamp DI. DI, disposition index; IGT, impaired glucose tolerance; MCRI, metabolic clearance rate of insulin; NGT, normal glucose tolerance; PIS, peripheral insulin sensitivity; T2D, type 2 diabetes.

Figure 1 illustrates the differences in MCRI by glycemic status overall and by race. In the total cohort, MCRI was lower in the presence of type 2 diabetes versus NGT and IGT, without a significant difference between IGT and NGT (Figure 1A). MCRI was lower in Black versus White youth (Figure 1B), driven by the lower MCRI in Black youth with IGT and type 2 diabetes without a significant difference between Black and White youth with NGT (Figure 2A). These results remained consistent after adjustment for age, sex, Tanner stage, and BMI (Figure 1A,B).

### Clamp‐derived metabolic characteristics by race within each glycemic status

Figure 2 depicts clamp‐derived metabolic characteristics by race in youth with NGT, IGT, and type 2 diabetes. In youth with NGT, MCRI, fasting insulin, and PIS were not different between Black and White youth, but first‐phase insulin and DI were significantly higher in Black youth. On the other hand, in youth with IGT and type 2 diabetes, whereas fasting insulin and PIS were not different between Black and White youth, MCRI was lower and first‐phase insulin was higher in Black youth, with no difference in DI. In each racial and ethnic group, there was a significant decrease in DI ~79% in Black youth and ~78% in White youth from NGT to type 2 diabetes (Figure S1). The finding remained consistent following adjustment for covariates.

### Relationship of MCRI to metabolic characteristics and adiposity measures in youth with obesity

**FIGURE 3:**
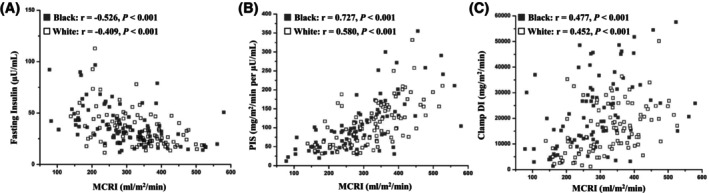
Correlations of MCRI with (A) fasting insulin, (B) PIS, and (C) clamp DI (untransformed data displayed) in Black and White youth with obesity. DI, disposition index; MCRI, metabolic clearance rate of insulin; PIS, peripheral insulin sensitivity.

Figure 3 shows the correlation of MCRI with fasting insulin concentration (Figure 3A), PIS (Figure 3), and DI (Figure 3C) in Black and White youth separately. In the total cohort, and in each racial and ethnic group, MCRI correlated negatively with fasting insulin concentration and positively with PIS and DI. Table S1 depicts the correlation of MCRI with adiposity measures, including weight, BMI, BMI *z* score, fat mass, percent body fat, VAT, leptin, and leptin‐adiponectin ratio, all of which were significantly and inversely correlated with MCRI, whereas adiponectin was positively correlated.

## DISCUSSION

The present investigation demonstrates that, in youth with obesity, 1) MCRI is significantly lower in the presence of type 2 diabetes versus IGT and versus NGT, with no difference between the latter two; 2) MCRI is lower in Black versus White youth driven by the significant difference in MCRI in the presence of IGT and type 2 diabetes and not in NGT; and 3) MCRI is negatively correlated with fasting insulin and adiposity measures and positively correlated with PIS and β‐cell function.

Plasma insulin concentrations are dependent on two separate but interrelated dynamics, i.e., insulin secretion by the pancreatic β cells and MCRI. Whereas the latter has been extensively studied in adults for some time [22, 23], the pediatric literature is sparse. It is well established that obesity is associated with decreased insulin clearance in adults [23, 24, 25] and in children [26, 27, 28, 29]; however, the impact of dysglycemia (IGT and/or type 2 diabetes) remains somewhat controversial. In the current study of youth aged 10 to 20 years with obesity, those with type 2 diabetes had 45% lower MCRI than their normoglycemic peers. This may represent a compensatory mechanism to maintain quasi‐hyperinsulinemia to counterbalance the defect in β‐cell function, ~78% decline in DI, and ~46% decline in first‐phase insulin secretion in the presence of type 2 diabetes (Table 1). Our finding, although consistent with observations in adults without obesity in whom MCRI was lower in the presence of type 2 diabetes, differs from that in adults with obesity, in whom MCRI did not decrease any further in the transition from NGT to type 2 diabetes [24]. This contrasting observation in MCRI between adults and youth with obesity and dysglycemia is consistent with the RISE results that youth with obesity and recently diagnosed diabetes and IGT, compared with their adult counterparts, had lower MCRI, assessed by the ratio of fasting C‐peptide to fasting insulin, in the presence of greater β‐cell dysfunction [6]. Given the youth–adult contrast in insulin dynamics, specific characterization of MCRI changes along with varying degrees of adiposity and glucose tolerance and by race and ethnicity in youth is critical.

Our current observation of significantly reduced MCRI in the presence of type 2 diabetes versus NGT or IGT and in Black versus White youth with obesity advances the limited pediatric literature. Several studies have assessed insulin clearance in youth, mainly using the C‐peptide‐to‐insulin ratio with no or limited information on glycemia‐related differences. A multiethnic (i.e., White, Black, and Hispanic) observational study [30] of 110 youth aged 14 to 21 years with normal weight and obesity showed that youth with obesity and IGT, as well as insulin‐resistant youth with obesity and NGT, have lower fractional hepatic MCRI than normal‐weight youth and insulin‐sensitive youth with obesity. In contrast, we observed no difference in MCRI between NGT (*n* = 138) versus IGT (*n* = 36) in the current study. Considering that our youth participants had relatively higher degrees of obesity (and higher visceral fat), and as we did not include a Hispanic group, it is difficult to compare or contrast our findings with those of the aforementioned study. A more recent cohort of 973 non‐Hispanic White youth with obesity (age range 3–17.9 years) showed that the development of prediabetes is negatively associated with MCRI measured by the ratio of the incremental areas under the curve (AUC) of C‐peptide to AUC of insulin during a 2‐h OGTT [31]. Despite their large cohort, the study population was heterogeneous (i.e., 35% were prepubertal children, and 57% had mild‐to‐severe liver steatosis disease), thereby hindering our understanding of exclusive effects of glycemia on MCRI in youth with obesity. More importantly, given that minorities including Black youth are disproportionately impacted by dysglycemia, the race‐ and ethnicity‐specific analysis in MCRI adds and supplements pediatric evidence.

In a study of Black and White children aged 8 to 14 years among whom only 25% had obesity, hepatic insulin extraction, calculated as the incremental C‐peptide‐to‐insulin molar ratio during an intravenous glucose tolerance test, was lower in Black versus White youth [32]. However, there was no information regarding the glucose tolerance status of the participants. In another very small study of 15 Black and 14 White youth with Tanner stages I to III, hepatic insulin extraction, estimated by C‐peptide and insulin modeling, was lower in Black versus White youth, but, again, glucose tolerance status was not specified [33]. Similarly, Piccinini et al. [34] found 74% lower hepatic MCRI (estimated by C‐peptide and insulin modeling) in Black versus White youth aged 7 to 13 years with normal weight [34], but this study also lacked information on glucose tolerance state. In obesity, a multiethnic cohort of 632 youth (aged 7–18 years) demonstrated that basal and stimulated MCRI, determined by the ratio between insulin secretion rate and plasma insulin levels at fasting and during a 3‐h OGTT, is lower in Black versus White youth, with similar MCRI levels observed between White and Hispanic youth [35]. However, the difference in MCRI was not tested by glycemic status. In contrast to our current findings of no difference between Black and White youth in MCRI in the group with NGT per se, a previous study of ours in normal‐weight, normoglycemic prepubertal children revealed that MCRI was lower in Black versus White youth (mean [SE] 14.5 [0.7] versus 16.8 [0.9] mL/m^2^/min; *p* = 0.037), the former of whom also had ~22% lower insulin sensitivity than their White peers [9]. The discrepancy between these two studies is likely driven by obesity and advanced puberty in the present cohort, suggesting that racial and ethnic contrast in MCRI could be overshadowed by obesity in the normoglycemic state. However, these racial and ethnic differences resurface when dysglycemia develops, potentially as a compensatory mechanism for the heightened insulin resistance and impaired β‐cell function in high‐risk Black youth. As can be deduced from Figure S1, in Black youth, the progressive decline from NGT to IGT to type 2 diabetes in MCRI (Figure S1A) is paralleled with a progressive increase in fasting plasma insulin concentrations (Figure S1B) unlike that in White youth.

Concomitant with lower MCRI in youth with type 2 diabetes, adiponectin was significantly lower in youth with type 2 diabetes compared with NGT, consistent with our earlier findings [8]. Additionally, adiponectin correlated significantly and positively with MCRI in the total cohort and separately in Black and White youth (Table S1). Even though correlations do not imply causations, it is possible that adiponectin may play a role in MCRI through its insulin‐sensitizing capacity.

To our knowledge, there are limited data regarding racial and ethnic differences in MCRI in youth [36, 37]. One study included a cohort of 709 children and adolescents with a very wide age range, i.e., 4 to 20 years, which measured MCRI by the molar ratio of AUC of insulin to C‐peptide during a 3‐h OGTT in conjunction with glucose tolerance status (NGT and IGT only) [36]. Weiss et al. [36] observed 22% lower MCRI in Black versus White youth with IGT, consistent with our findings of 20.5% and 26.5% lower MCRI in Black youth with IGT and type 2 diabetes, respectively. However, contrary to our findings, they showed that Black youth with NGT have lower MCRI compared with White youth. These differing findings could be explained by the inclusion of prepubertal children in the former study (21.3% of participants were prepubertal) compared with ours, in which all were pubertal. In addition, the Treatment Options for Type 2 Diabetes in Adolescents and Youth (TODAY) study of 640 youth (aged 10–18 years) with obesity and type 2 diabetes recently showed results consistent with ours, i.e., that Black versus White youth had 23.8% lower fasting and 24.4% lower 2‐h OGTT MCRI (calculated from the molar ratio of fasting C‐peptide to insulin or the molar ratio of AUC of insulin to C‐peptide during a 2‐h OGTT, respectively) [37]. It is notable that the TODAY study (90.2% of participants were in Tanner stages 4–5), similar to our study (91.7% in Tanner stages 4–5), included the predominant proportion of youth with late‐stage puberty. However, as mentioned earlier, the TODAY study was limited to youth with type 2 diabetes and did not include those with NGT to allow for the investigation of whether racial and ethnic differences exist between Black and White youth with NGT. Given that pubertal progression is associated with impairment of MCRI in youth with obesity [31], its interaction with race and ethnicity should be further examined.

In our study, the lower MCRI in Black versus White youth with dysglycemia (Figure 2A) occurred together with higher first‐phase insulin in Black youth, i.e., ~170% in the presence of IGT and ~275% in the presence of type 2 diabetes (Figure 2D). In addition, MCRI positively correlated with PIS and clamp DI and negatively with fasting insulin in both Black and White youth (Figure 3). As such, our data suggest that there is a strong homeostatic interplay among insulin clearance, secretion, and action, all contributing to glucose homeostasis. Note that clamp DI was similar between Black and White youth with IGT and type 2 diabetes in the present study, and we postulated that severe impairment of MCRI contributes to hyperinsulinemia at fasting and during the clamp, thereby paradoxically compensating for severe β‐cell dysfunction in Black versus White youth with IGT and type 2 diabetes. However, prolonged hyperinsulinemia would further exacerbate insulin resistance and amplify pancreatic β‐cell dysfunction, exposing Black youth who are at higher risk of developing type 2 diabetes and its complications [38]. Finally, the negative correlations of MCRI with BMI, percent body fat, and VAT (Table S1) are consistent with previous literature [31, 39].

The strengths of the present investigation include the following: 1) a robust characterization of racial and ethnic differences in insulin dynamics, including MCRI, insulin sensitivity, and β‐cell function (all measured by the clamp methodology), in youth with obesity across the spectrum of glucose tolerance, including NGT, IGT, and type 2 diabetes; and 2) a well‐balanced study population according to sex and race and ethnicity (i.e., Black and White). A potential perceived limitation would be that youth with type 2 diabetes were on diverse treatments, including metformin, insulin, metformin plus insulin, or lifestyle modification. Although this could modify insulin metabolism, a variety of treatments is unavoidable as, when a youth is diagnosed with type 2 diabetes, treatment is initiated, and therapeutic methods are determined by the severity of hyperglycemia and the choice of a provider in a clinical setting. Therefore, to minimize this effect, in youth with type 2 diabetes, metformin and long‐ or intermediate‐acting insulin were discontinued 48 h prior to the clamp, as has been reported previously [13, 14]. Compared with our group with NGT, the limited number of youth with dysglycemia (i.e., 36 with IGT and 32 with type 2 diabetes) may reduce our analytical power for detecting differences in MCRI and its related variables by glucose tolerance. However, it should be noted that our proportion of each glycemic group falls in the general prevalence of glycemia in youth [40]. Our study population may have limited generalizability of the findings due to the lack of racial and ethnic diversity, as only Black and White youth were included, which limits the generalizability to other populations with different racial and ethnic backgrounds (i.e., American Indian/Alaska Native, Asian, and Hispanic individuals). With regards to autoantibody testing, we did not measure insulin or zinc transporter 8 (ZnT8) antibodies, the likelihood of which being positive is low. In the TODAY study, based on a provider's diagnosis of type 2 diabetes, only 2 out of 687 youth (0.29%) were ZnT8‐positive at screening among those with negative glutamic acid decarboxylase and insulinoma‐associated protein‐2 antibodies [41]. In another study, insulin antibodies occurred at a relatively low proportion (i.e., 2.22%, 11/495) in adults who were clinically diagnosed with type 2 diabetes based on fasting, random, or 2‐h plasma glucose concentrations during a 2‐h OGTT [42]. Another limitation of the study is that, due to the nature of the cross‐sectional design, we were unable to extrapolate causal relationships among reduced MCRI, hyperinsulinemia, and insulin resistance. Additionally, we did not conduct a somatostatin infusion during a hyperinsulinemic‐euglycemic clamp due to our target population (i.e., adolescents); therefore, we cannot fully attribute the suppression of endogenous insulin secretion to the insulin infusion alone [43]. Moreover, the present analyses did not include C‐peptide concentrations during the hyperinsulinemic‐euglycemic clamp to verify the suppression of endogenous insulin secretion; therefore, our clamp‐derived MCRI may slightly underestimate the true values. However, given that the contribution of residual insulin secretion to steady‐state plasma insulin is expected to be minimal under hyperinsulinemic infusion conditions, we believe that there will be a minimal impact on our findings. In addition, we did not apply mathematical modeling that could differentiate intra‐ versus extrahepatic MCRI [31, 36]. Finally, we measured β‐cell function by clamp experiments, which have been considered gold‐standard measures but do not allow for measuring incretin effect. Given that incretin effect has been linked to physiological capacity of pancreatic β‐cell response [44], it would be worth investigating the potential relationships among MCRI, incretin effect, and β‐cell function.

In summary, our data reveal that, contrary to our previous observations in prepubertal children with normal weight [9], there is no significant racial and ethnic difference (i.e., in the context of Black vs. White) in MCRI among pubertal youth with obesity and NGT. However, in pubertal youth with obesity and IGT and type 2 diabetes, we found significantly lower MCRI in Black versus White youth, signifying its contribution to compensatory fasting hyperinsulinemia in the presence of β‐cell dysfunction. Taken together, our data suggest that the interplay among insulin clearance, action, and secretion plays a pivotal role in glucose homeostasis in youth with obesity.

## AUTHOR CONTRIBUTIONS

Wonhee Cho, Joon Young Kim, and Silva Arslanian designed the study, analyzed data, and wrote the manuscript. Fida Bacha, Hala Tfayli, and SoJung Lee contributed data. Fida Bacha, Hala Tfayli, SoJung Lee, and Sara F. Michaliszyn reviewed the manuscript. Wonhee Cho, Fida Bacha, Hala Tfayli, SoJung Lee, Sara F. Michaliszyn, Joon Young Kim, and Silva Arslanian approved the manuscript in its final version. Silva Arslanian is the guarantor of this work and, as such, had full access to all the data in the study and takes responsibility for the integrity of the data and the accuracy of the data analysis.

## FUNDING INFORMATION

This study was supported by National Institute of Child Health and Human Development grants K24‐HD01357 and R01‐HD27503 to Silva Arslanian, National Center for Advancing Translational Sciences Clinical and Translational Science Award UL1TR000005, and National Center for Research Resources grant UL1RR024153 to the General Clinical Research Center.

## CONFLICT OF INTEREST STATEMENT

Silva Arslanian receives research funding from Novo Nordisk A/S and Eli Lilly and Company and consulting fees from Societe des Produits Nestlé (Nestlé Products Company) and participates on a data safety monitoring board or advisory board from Novo Nordisk A/S, Eli Lilly and Company, AstraZeneca plc, and Boehringer Ingelheim. The other authors declared no conflicts of interest.

## Supporting information


**FIGURE S1.** Differences in clamp‐derived metabolic characteristics across glucose tolerance category (NGT, IGT, and type 2 diabetes) in each race: (A) MCRI, (B) fasting insulin, (C) peripheral insulin sensitivity, (D) first‐phase insulin, and (E) clamp DI.

**TABLE S1.** Correlation of MCRI with adiposity measures in youth with obesity.

## Data Availability

All data supporting the study's findings are available within the study. For any additional questions, please feel free to reach out to the corresponding author via email.
