# Supplementary material for: Metabolic clearance rate of insulin across the glucose tolerance spectrum by race and ethnicity in youth with obesity
Source: Obesity (Silver Spring). 2025 Jun 5;33(7):1365–74. doi: 10.1002/oby.24317 (PMC12210107; doi:10.1002/oby.24317)
Supplement: Supplementary file 1 — FIGURE S1. Differences in clamp‐derived metabolic characteristics across glucose tolerance category (NGT, IGT, and type 2 diabetes) in each race: (A) MCRI, (B) fasting insulin, (C) peripheral insulin sensitivity, (D) first‐phase insulin, and (E) clamp DI. [file OBY-33-1365-s001.pdf]

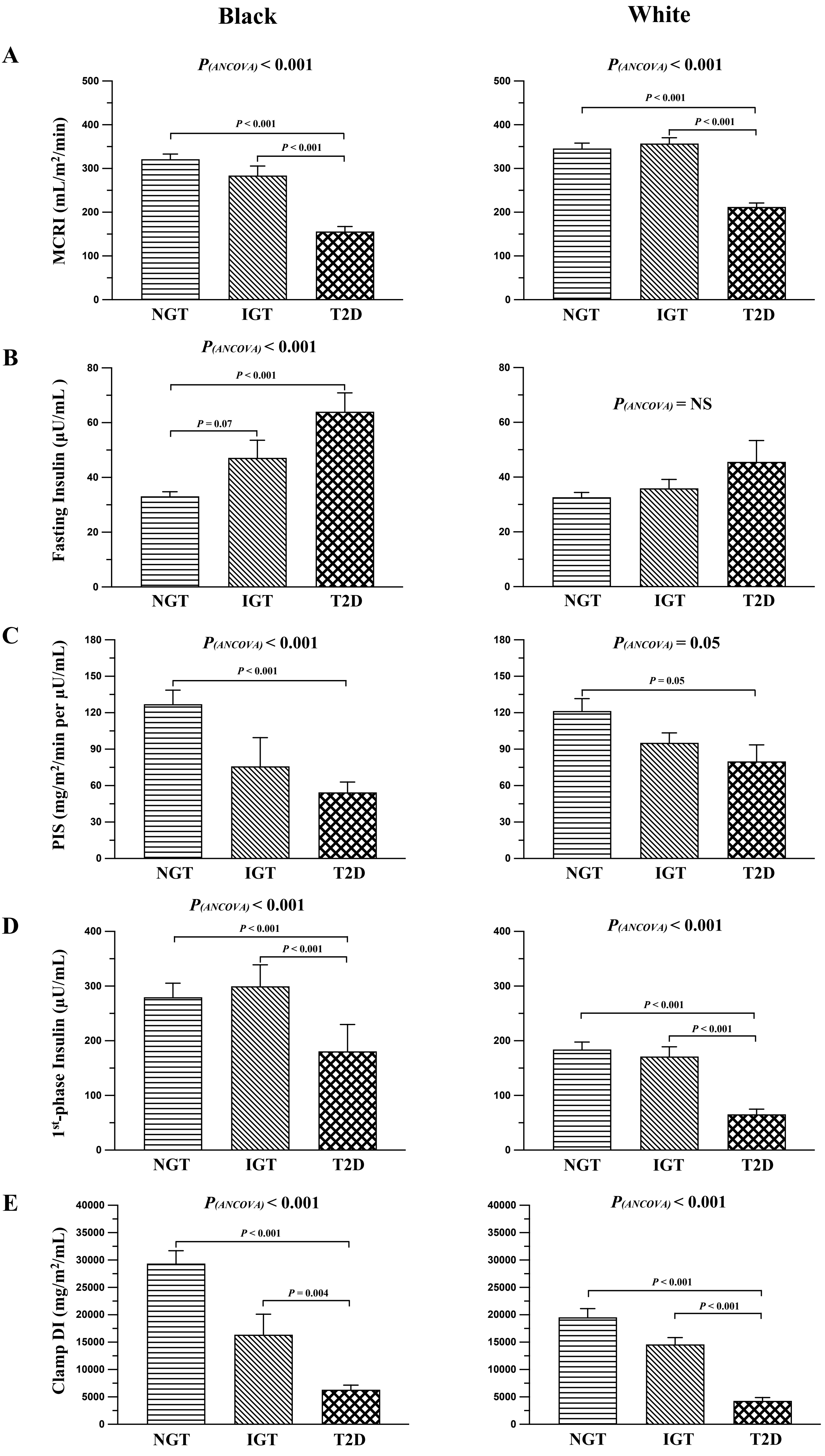

Figure S1. Differences in clamp-derived metabolic characteristics across glucose tolerance category (NGT, IGT, and type 2 diabetes) in each race: (A) MCRI, (B) fasting insulin, (C) peripheral insulin sensitivity, (D) first-phase insulin, and (E) clamp DI.
