# Supplementary material for: Metabolic clearance rate of insulin across the glucose tolerance spectrum by race and ethnicity in youth with obesity
Source: Obesity (Silver Spring). 2025 Jun 5;33(7):1365–74. doi: 10.1002/oby.24317 (PMC12210107; doi:10.1002/oby.24317)
Supplement: Supplementary file 2 — TABLE S1. Correlation of MCRI with adiposity measures in youth with obesity. [file OBY-33-1365-s002.docx]

**Table S1.** Correlation of MCRI with adiposity measures in youth with obesity

|  | **Total**  **(N=206)** | **Black**  **(n=95)** | **White**  **(n=111)** |
| --- | --- | --- | --- |
| Weight | -0.319** | -0.321** | -0.327** |
| BMI | -0.422** | -0.437** | -0.401** |
| BMI-z score | -0.287** | -0.206* | -0.343** |
| Fat Mass | -0.357** | -0.396** | -0.337** |
| Percent Body Fat | -0.284** | -0.307** | -0.312** |
| VAT | -0.297** | -0.348** | -0.418** |
| Leptin | -0.295** | -0.351** | -0.243* |
| Adiponectin | 0.337** | 0.448** | 0.215* |
| Leptin-adiponectin ratio | -0.436** | -0.590** | -0.290** |
| **P <* 0.05, ***P <* 0.001  VAT, Visceral adipose tissue | | | |
